# Entheseal Doppler signals in ultrasound are associated with vasodilator drugs and age in patients with radiographic axial spondyloarthritis

**DOI:** 10.1186/s13075-025-03614-8

**Published:** 2025-07-14

**Authors:** Magnus Hallström, Anna Deminger, Caroline Feldthusen, Erik Hulander, Mats Geijer, Eva Klingberg, Helena Forsblad-d’Elia

**Affiliations:** 1 Department of Rheumatology and Inflammation Research, Institute of Medicine, University of Gothenburg, Box 480, Gothenburg, 405 30 Sweden; 2 Department of Rheumatology, Region Västra Götaland, Sahlgrenska University Hospital, Gothenburg, Sweden; 3 Department of Occupational Therapy and Physiotherapy, Region Västra Götaland, Sahlgrenska University Hospital, Gothenburg, Sweden; 4 Clinical Nutrition Unit, Region Västra Götaland, Sahlgrenska University Hospital, Gothenburg, Sweden; 5 Department of Radiology, Institute of Clinical Sciences, University of Gothenburg, Gothenburg, Sweden; 6 Department of Radiology, Region Västra Götaland, Sahlgrenska University Hospital, Gothenburg, Sweden; 7 Department of Clinical Sciences, Lund University, Lund, Sweden

**Keywords:** Axial spondyloarthritis, Ankylosing spondylitis, Enthesopathy, Doppler ultrasonography, Diagnostic imaging

## Abstract

**Background:**

The ability of modern ultrasound machines to detect signs of enthesitis has increased, yet there is a lack of studies on patients with long-standing radiographic axial spondyloarthritis (r-axSpA). Hence, we aimed to investigate the prevalence and clinical significance of Doppler signals indicative of inflammation in peripheral entheses of patients with long-standing disease.

**Methods:**

Patients fulfilling the modified New York criteria for ankylosing spondylitis were included in this cohort study. Peripheral entheses were examined clinically and the presence of focal pain was self-reported on a mannequin. Ultrasound examination of 1692 entheses was performed. Doppler signals were graded from 0 to 3 using color Doppler ultrasound and Smooth Microvascular Imaging. Multivariable linear regression was used to explore factors influencing Doppler signals.

**Results:**

One hundred and forty-one patients were included with, age (mean (SD)) 60 (12) years, symptom duration 34 (12) years, males 57%, and HLA-B27 86%. Overall, 21.3% of patients presented with ≥ 1 active ultrasound enthesitis (Doppler signals combined with hypoechoic tissue). In 4.3% of patients these findings were tender on palpation. Isolated Doppler signals were found in 89.4–97.1% of patients, with the highest mean Doppler grades in the triceps entheses (0.88), and the lowest in the Achilles tendons (0.28). In multivariable linear regression analysis, age (B (95% CI)) (0.01 (0.00; 0.01), *p* = 0.004), daily NSAIDs (0.15 (0.00; 0.30), *p* = 0.048), vasodilator drugs 0.16 (0.01; 0.32, *p* = 0.041), but not AS disease activity score, were associated with total Doppler scores.

**Conclusion:**

The prevalence of asymptomatic entheseal ultrasound Doppler findings was overall high. The use of vasodilator drugs and higher age increased the Doppler scores. No association between disease activity and Doppler scores was found in patients with long-standing disease.

**Supplementary information:**

The online version contains supplementary material available at 10.1186/s13075-025-03614-8.

## Background

Radiographic axial spondyloarthritis (r-axSpA) is an inflammatory rheumatic disease that primarily affects the sacroiliac joints and spine. The onset of symptoms is mostly before 40 years of age and there is a male predominance [1]. A key feature of the disease is inflammation that emerges at the entheses where tendons, ligaments, and joint capsules attach to the bone [2]. Though insertional tendinopathy also occurs in persons without disease, enthesitis is considered an expression of axSpA, as reflected by the Assessment of SpondyloArthritis international Society (ASAS) classification criteria for axSpA [3]. In addition, a clinical enthesitis index, the Maastricht Ankylosing Spondylitis Enthesitis Score (MASES), is included in the ASAS core outcome set for axSpA [4, 5]. Clinical assessment of enthesitis is demanding as distinguishing between inflammatory and non-inflammatory tenderness can be difficult [6, 7]. Therefore, ultrasound (US) has been increasingly used in rheumatology care, and a consensus-based definition of US-detected enthesitis has been published [8]. However, entheseal findings indicative of inflammation are also reported in healthy individuals and are related to age, sex, body mass index (BMI), and physical activity, which pose a challenge for sonographers [9]. Moreover, subclinical signs of enthesitis have been reported as more prevalent in individuals with axial spondylitis, early psoriatic arthritis (PsA) and in those with inflammatory bowel disease (IBD) compared to healthy controls [10–13]. Neovascular entheseal changes, referred to as Doppler signals have been found less prevalent in healthy subjects [9], and excelled as the inflammatory imaging lesion that best agrees with clinical examination in a recent study on patients with SpA [14].

The treatment arsenal of r-axSpA has grown in recent years, which stresses the importance of accurately assessing disease activity. Furthermore, modern US machines are increasingly available in rheumatology practice and influence clinical decision-making in daily care. Since there are multiple confounders, and as the sensitivity of US equipment increases, there is a rationale for investigating the significance of findings indicative of active inflammation. In addition, studies on subclinical signs of enthesitis in long-standing r-axSpA are lacking.

The primary aim of this study was to investigate the impact of disease activity, along with other potential influencing factors, on a composite Doppler score based on US examination of peripheral entheses in patients with long-standing r-axSpA. Secondary aims were to describe the prevalence of Doppler signals in peripheral entheses and their relationship with clinical signs and symptoms of enthesitis, including established clinical enthesitis indices. A further aim was to examine whether Doppler signals in specific pairs of entheses showed a higher association with disease activity than others.

## Methods

### Patients

**Fig. 1:**
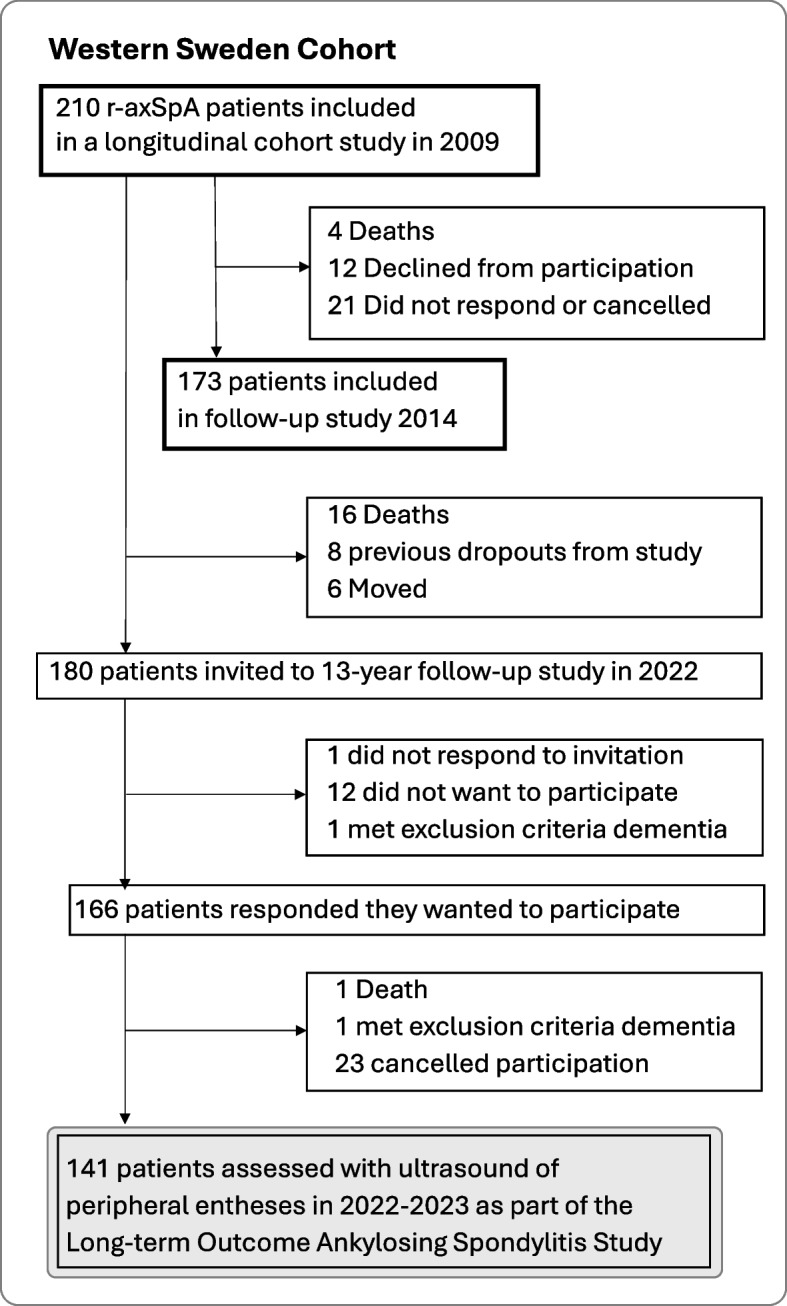
Flowchart of the inclusion process of patients with radiographic axial spondyloarthritis. Legend: *r-axSpA* radiographic axial spondyloarthritis

Patients in western Sweden fulfilling the modified New York criteria for ankylosing spondylitis (AS) (ICD-10 M45.9) were invited as part of the longitudinal Long-term Outcome AS (LOAS) study [15]. Exclusion criteria were dementia, pregnancy, difficulty in understanding the Swedish language, and to homogenize the cohort, patients with inflammatory bowel disease and psoriasis. All patients were initially recruited in 2009, as previously described, and of the originally 210 patients, 141 were included in the current 13-year follow-up study [16]. The inclusion process of the LOAS cohort is shown in Fig. 1. Written informed consent was obtained from all patients. The study was approved by the Swedish ethical review board (597–08, 2021–03484) and performed in compliance with the Declaration of Helsinki.

### Ultrasound examination

Ultrasound examinations were performed at the same study visit as blood sampling, clinical examinations, and the patient-reported outcome measures. All participants'entheses were examined using US (Aplio i700, 18L7 linear transducer, Canon Medical Systems Corp., Otawara, Japan). All examinations were performed in the same spacious, well-ventilated facility by one US-profiled rheumatologist with more than five years of experience (MH). Throughout the study, the Doppler settings were fixed and optimized to enable visualization of low velocity flow. Two different Doppler applications were used as shown in Supplementary Figure S1, Additional File 1 (color Doppler ultrasound (CDU); color frequency 7.5, scale 4 cm/s, color gain 25 and smooth microvascular imaging (SMI); frequency 7.5, scale 2.2 cm/s, color gain 44). CDU was chosen instead of power Doppler (PD) to ensure the most sensitive application [17]. During the assessment of vascular changes, position adjustments were made to ensure relaxed tendinous tissues. In accordance with a previous consensus statement, the enthesis was defined as the area < 2 mm from the bony cortex (A) [8]. Findings slightly proximal to this area were also registered and referred to as outside the enthesis (B), as shown in Supplementary Figure S2, Additional File 1. The following entheses were assessed bilaterally: triceps distal enthesis, common extensor origin at the lateral humeral epicondyle, quadriceps distal enthesis, proximal patellar ligament origin, distal patellar ligament insertion, and the Achilles enthesis. The plantar fascia was evaluated for structural lesions but because of the dense plantar fat pad, Doppler assessment was excluded. Doppler findings were semi-quantitatively scored from 0 to 3 according to recommendations of the Outcome Measures in Rheumatology (OMERACT) US working group [18]. The sonographer was blinded to background medication and the presence of focal tenderness. Before the study start, efforts were made to ensure strong reliability of the US examinations, including several seminars focusing on enthesitis scoring according to OMERACT recommendations [18]. To evaluate intra-observer reliability, we focused exclusively on Doppler grading and enthesophyte measurements, given the study aim and the high prevalence of these lesions. Eighty pre-recorded, unlabeled images from the study sample were scored and re-scored with an interval of three months.

### Ultrasound variables

For the primary analyses, a composite total Doppler score from 0 to 36 was calculated by summarizing Doppler grades in the entheses (A) of all examined sites. When evaluating pairs of tendons in relation to disease activity, mean Doppler grade in the enthesis (A) was used. In the analyses of the relationship between clinical signs and symptoms, maximum Doppler grade at each anatomical location was used; measured in the enthesis, or outside the enthesis (A or B). For sensitivity analyses, a composite total Doppler score from 0 to 72 was also calculated based on Doppler grades in and outside the entheses (A + B) of all examined sites.

Both CDU and SMI data was used for comparison and as sensitivity analyses. In the examination of the relationship between Doppler findings and clinical signs and symptoms, the CDU was used exclusively because it is more commonly available compared to the SMI application. Tendon insertions were measured, hypoechogenicity and bursitis were reported as present or not. Active US enthesitis was defined as the presence of entheseal Doppler signals and hypoechoic tendinous tissue < 2 mm from cortical bone. In case of indeterminate echogenic status due to tears or large enthesophytes, Doppler-positive thickened insertion < 2 mm from bone was also considered as active US enthesitis. Enthesophytes, calcifications, and erosions were counted and measured for the calculation of a summarized chronicity score ranging from 0 to 210, as described previously [19].

### Physical examination and patient-reported outcome measures

All participants filled out a questionnaire regarding the level of physical activity, occupational physical activity workload, and current medication. Physical activity was reported by answering categorical questions regarding hours of vigorous-intensity physical activity per week and registered on a scale from 0 to 5 [20]. Zero minutes of vigorous physical activity was graded as 0, while more than two hours per week gave a maximum score of 5. To estimate occupational physical activity workload, participants were asked to describe their current and previous occupations as mostly sedentary or mostly walking with or without heavy lifting on a scale from 1 to 4. Weight and length were measured at the study visits, and AS disease activity score (ASDAS) was assessed [21]. For longitudinal data, mean values of BMI and ASDAS were calculated based on data from the present and previous study visits in 2009 and 2014. A non-steroidal anti-inflammatory drugs (NSAID) index was calculated based on use during the present calendar year at the study visit [22]. After ultrasound examination of the entheses, patients were assessed clinically for 66/68 joint count and enthesitis by the same experienced rheumatologist (MH), including the Maastricht AS Enthesitis Score (MASES), Leeds Enthesitis Index (LEI), and the Spondyloarthritis Research Consortium of Canada (SPARCC) enthesitis index [5, 23, 24]. MASES, LEI, and SPARCC were scored from 0 to 13, 0 to 6, and 0 to 16, respectively. A positive clinical enthesitis test was defined by tenderness on palpation. The patients were also asked to report pain with a duration longer than three months on a mannequin. Asymptomatic entheses were defined by the absence of reporting focal pain on the mannequin and no tenderness on palpation.

### Laboratory tests and radiographs

Blood samples were drawn at the study visit to analyze inflammatory markers, including high-sensitivity C-reactive protein (hs-CRP), erythrocyte sedimentation rate (ESR), and leukocyte differential count using standard laboratory techniques. Longitudinal data on CRP and ESR were collected from medical records between 2009 −2022. The first measurement of these biomarkers in each calendar year during the period was registered, and a mean value was calculated based on all available measurements for each patient. Lateral radiographs of the spine were acquired according to the study protocol. Scoring of modified Stoke Ankylosing Spondylitis Spinal Score (mSASSS) was performed by one experienced and previously reliability-tested musculoskeletal radiologist (MG) [25, 26].

### Statistics

Mean and standard deviation (SD) and median and quartile 1/quartile 3 were used to describe continuous variables according to distribution. The Wilcoxon signed-rank test was used to compare Doppler scores measured with CDU and SMI. Friedman’s test was used to investigate differences between non-normally distributed CDU grades measured in multiple different anatomical locations, followed by pairwise comparisons using the Wilcoxon signed-rank test with Bonferroni correction to adjust for multiple testing. The Mann–Whitney U-test was employed when comparing two independent groups regarding non-normally distributed CDU and SMI total scores. Intraclass correlation (ICC) coefficient was calculated using a two-way mixed effects model, absolute agreement, and average measures to assess intra-observer reliability of the sonographer. ICC values < 0.5 were regarded as poor, whereas values between 0.5 to 0.75, 0.75 to 0.9, and values > 0.9 indicated moderate, good, and excellent reliability, respectively [27]. The Pearson correlation coefficient (*ρ*) was employed for linear correlations.

Multivariable linear regression analysis was used to investigate influencing factors of a composite total Doppler score measured with CDU and SMI. Standard method was applied, missing values were handled by pairwise exclusion, and regarding collinearity, *ρ* < 0.7 was accepted. Residual plots were assessed for heteroscedasticity, which was managed by log transforming the dependent variable of the models using log(Y + 1) to account for zero values. Independent variables were chosen based on hypothetical reasoning and previous reports in the literature. These covariates include age, BMI, male sex, ASDAS, ESR, vigorous physical activity, daily non-steroidal anti-inflammatory drugs (NSAIDs), biological disease-modifying anti-rheumatic drug (bDMARD) treatment, and vasodilator drugs. Vasodilator drugs were defined as calcium channel blockers (CCBs), glyceryl trinitrate, and buprenorphine; the latter because of the strong vasodilating side effect. Further models were performed to investigate long-term effects on entheseal Doppler scores, using longitudinal retrospective data on ASDAS, CRP, ESR, occupational physical activity workload, BMI, and entheses chronicity score as independent variables. Previous occupational physical activity workload was based on reported workload during the third to fifth decade of life.

As sensitivity analyses, the regression models were performed using a different outcome: total Doppler score calculated from Doppler measured both in and outside of the enthesis (A + B). To separate the effect of vasodilators from other antihypertensives, antihypertensive medication including angiotensin-converting enzyme (ACE) inhibitors, angiotensin receptor blockers (ARBs), and diuretics were adjusted for in an additional model. All analyses were performed using SPSS statistics version 29.0.1.1 (SPSS Inc., IBM Corp., Armonk, NY, USA). A *p*-value < 0.05 was considered statistically significant.

## Results

### Patient characteristics and entheseal US findings

As shown in Table 1, the presence of human leukocyte antigen (HLA)-B27 was 85.8%, the mean (SD) age was 60 (12) years, and the symptom duration was 34 (12) years. The mean (SD) ASDAS was 2.0 (0.8), and the mean LEI, MASES, and SPARCC scores were 0.3 (0.1), 1.3 (0.2), and 0.9 (0.1), respectively. BDMARDs were used by 35.0% of patients, and 19.1% used daily NSAIDs. Vasodilator drugs were used by 15.6% of the patients. Furthermore, 30 (21.3%) patients presented with ≥ one active US enthesitis (Table 2). In only 6 (4.3%) patients, these findings corresponded to tenderness on palpation. Regarding isolated Doppler signals, 97.2% and 89.4% of patients displayed at least one Doppler signal in examined entheses (A, Supplementary Fig S2, Additional File 1) when assessed by CDU and the SMI application, respectively. A statistically significant difference was observed between SMI and CDU, with a higher mean total Doppler score in the enthesis (A) when measured with CDU, 6.9 (5.9) compared to SMI, 5.9 (5.6) (*p* < 0.001). There was no difference between the applications when total Doppler scores outside the enthesis (B) were compared (*p* = 0.30).

**Table 1:** Characteristics of 141 patients with radiographic axial spondyloarthritis

	All, *n* = 141	Male, *n* = 81	Female, *n* = 60
Age, years	60 ± 12	59 ± 12	61 ± 12
BMI, kg/m^2^	26.3 ± 3.8	26.9 ± 3.5	25.5 ± 4.1
HLA-B27	121 (85.8)	75 (92.6)	46 (76.7)
Smoking daily	5 (3.5)	2 (2.5)	3 (5)
Symptom duration, years	34 ± 12^a^	34 ± 12^b^	35 ± 12
ASDAS, score	2.0 ± 0.8^a^	1.9 ± 0.8^b^	2.0 ± 0.8
BASDAI, score	3.4 ± 2.1	3.2 ± 2.1	3.6 ± 2.1
BASMI, score	3.2 ± 1.6	3.4 ± 1.8	3.0 ± 1.4
BASFI, score	2.5 ± 2.0	2.6 ± 2.0	2.4 ± 2.1
mSASSS, score	15.7 ± 20.3 6.0 (0.0, 24.0)	22.2 ± 23.0 13.0 (3.5, 34.5)	6.7 ± 10.9 1.0 (0.0, 10.3)^c^
Tender joint count 68, score	± 0.2 0.0 (0.0, 1.0)	0.7 ± 0.2 0.0 (0.0, 1.0)	1.4 ± 0.5 0.0 (0.0, 1.0)
Swollen joint count 66, score	0.0 ± 0.0 0.0 (0.0, 0.0)	0.0 ± 0.2 0.0 (0.0, 0.0)	0.0 ± 0.1 0.0 (0.0, 0.0)
LEI, score	0.3 ± 0.1 0.0 (0.0, 0.0)	0.2 ± 0.1 0.0 (0.0, 0.0)	0.4 ± 0.8 0.0 (0.0, 0.0)
MASES, score	1.3 ± 0.2 0.0 (0.0, 2.0)	0.8 ± 0.2 0.0 (0.0, 0.5)	2.1 ± 0.4 1.0 (0.0, 3.0)
SPARCC enthesitis index, score	0.9 ± 0.1 0.0 (0.0, 1.0)	0.5 ± 0.2 0.0 (0.0, 0.0)	1.4 ± 0.2 0.5 (0.0, 2.0)
High sensitivity C-reactive protein, mg/L	3.1 ± 0.4 1.7 (0.8, 3.9)	3.4 ± 0.6 1.7 (0.9, 4.0)	2.8 ± 0.4 1.8 (0.5, 3.5)
Erythrocyte sedimentation rate, mm/h	13.6 ± 1.0 10.0 (5.0, 18.0)^d^	13.1 ± 1.6 8.5 (4.0, 17.0)^b^	14.4 ± 1.1 12.0 (8.0, 19.0)^e^
Neutrophil count, 10^9^/L	3.5 ± 1.4	3.4 ± 1.3	3.7 ± 1.4
bDMARDS	49 (35.0)	33 (40,7)	16 (26.7)
csDMARD	24 (17.0)	12 (14.8)	12 (20.0)
Non-steroidal anti-inflammatory drugs, daily	27 (19.1)	16 (19.8)	11 (18.3)
Non-steroidal anti-inflammatory drugs, Index	16.1 ± 31.0 0.0 (0.0, 14.0)^e^	18.7 ± 33.3 0.0 (0.0, 22.0)^g^	12.5 ± 27.3 0.0 (0.0, 0.0)^c^
Level of physical activity, score	2.5 ± 1.8 3.0 (1.0, 4.0)	2.8 ± 1.8 3.0 (1.0, 5.0)	2.1 ± 1.6 2.0 (1.0, 3.0)
Occupational physical activity workload (current)	1.8 ± 0.9 2.0 (1.0, 2.0)^h^	1.7 ± 0.9 1.0 (1.0, 2.0)^i^	1.8 ± 0.9 2.0 (1.0, 2.0)^j^
Occupational physical activity workload (median previous)	2.2 ± 1.1 2.0 (1.0, 3.0)^k^	2.3 ± 1.2 2.0 (1.0, 4.0)^l^	2.1 ± 1.0 2.0 (1.0, 3.0)^m^
Vasodilator drugs	22 (15.6)	14 (17.2)	8 (13.3)
Amlodipine	16 (11.3)	10 (12.3)	6 (10.0)
Felodipine	4 (2.8)	2 (2.5)	2 (3.3)
Buprenorphine	1 (1.0)	1 (1.2)	0 (0)
Glyceryl trinitrate	1 (1.0)	1 (1.2)	0 (0)

^a^*n*=140,^b^*n* = 80, ^c^*n* = 58, ^d^*n* = 139, ^e^*n* = 59, ^f^*n* = 137, ^g^*n* = 79, *n*^h^ = 81, *n*^i^ = 46, *n*^j^= 35, *n*^k^ = 110, *n*^l^ = 66, *n*^m^ = 44

Values are mean ± standard deviation, median (quartile (Q)1, Q3), or n (%). *BMI* body mass index, *HLA-B27* human leukocyte antigen B27, *ASDAS* Ankylosing Spondylitis Disease Activity Score, *BASDAI* Bath Ankylosing Spondylitis Disease Activity Index, *BASMI* Bath Ankylosing Spondylitis Metrology Index, *BASFI* Bath Ankylosing Spondylitis Functional Index, *mSASSS* Modified Stoke Ankylosing Spondylitis Spinal Score, *LEI* Leeds enthesitis index, *MASES* Maastricht AS Enthesitis Score, *SPARCC* Spondyloarthritis Research Consortium of Canada, *bDMARD* biological disease-modifying anti-rheumatic drug, *cs* conventional synthetic, *level of physical activity* hours of vigorous-intensity physical activity per week

**Table 2:** Prevalence of entheseal ultrasound findings

	All,*n* = 141	Male, *n* = 81	Female, *n* = 60
CDU positive active US enthesitis (≥ 1 enthesis)	30 (21.3)	21 (25.9)	9 (15)
Active US enthesitis tender on palpation (≥ 1 enthesis)	6 (4.3)	5 (6.2)	1 (1.7)
Entheseal CDU/SMI (A) (≥ grade 1 in ≥ 1 enthesis)	137 (97.2)/ 126 (89.4)	77 (95.1)/ 72 (88.9)	60 (100)/ 54 (90.0)
Achilles bursitis ± positive CDU/SMI	9 (6.4)	3 (3.7)	6 (10)
Hypoechoic lesions (A) ± CDU/SMI (≥ 1 enthesis)	11 (7.8)	7 (8.6)	4 (6.7)
Doppler score upper extremity (CDU in A) (0–12)	3.1 ± 2.3 2.0 (1.0, 5.0)	2.7 ± 2.3 2.0 (1.0, 4.0)	3.6 ± 2.3 3.0 (2.0, 5.0)
Doppler score upper extremity (SMI in A) (0–12)	2.3 ± 2.2 2.0 (1.0, 4.0)	2.1 ± 2.2 1.0 (0.0, 4.0)	2.6 ± 2.2 2.0 (1.0, 4.0)
Doppler score lower extremity (CDU in A) (0–24)	3.8 ± 4.3 2.0 (1.0, 5.5)	3.7 ± 4.5 2.0 (0.5, 5.0)	3.9 ± 4.0 3.0 (1.0, 7.0)
Doppler score lower extremity (SMI in A) (0–24)	3.5 ± 4.1 2.0 (0.0, 5.5)	3.5 ± 4.3 2.0 (0.0, 4.5)	3.6 ± 3.8 2.0 (0.0, 6.8)
Total Doppler score in entheses (CDU in A) (0–36)	6.9 ± 5.9 6.0 (2.0, 10.0)	6.5 ± 6.2 4.0 (2.0, 9.0)	7.5 ± 5.6 7.0 (2.3, 11.0)
Total Doppler score in entheses (SMI in A) (0–36)	5.9 ± 5.6 4.0 (1.0, 9.0)	5.7 ± 5.9 4.0 (1.0, 8.0)	6.1 ± 5.2 4.5 (2.0, 10.0)
Total Doppler score outside the entheses (CDU in B) (0–36)	3.0 ± 4.2 2.0 (0.0, 5.0)	3.2 ± 4.8 2.0 (0.0, 4.0)	2.9 ± 3.3 1.0 (0.0, 5.8)
Total Doppler score outside the entheses (SMI in B) (0–36)	3.4 ± 4.3 2.0 (0.0, 5.0)	3.6 ± 4.9 2.0 (0.0, 5.0)	3.2 ± 3.5 2.0 (0.0, 5.0)
Total Doppler score in and outside the entheses (CDU in A + B) (0 −72)	9.9 ± 9.3 7.0 (3.0, 14.0)	9.6 ± 10.2 6.0 (3.0, 13.0)	10.4 ± 8.0 8.0 (4.0, 18.8)
Total Doppler score in and outside the entheses (SMI in A + B) (0 −72)	9.3 ± 9.3 6.0 (2.5, 14.0)	9.3 ± 10.2 6.0 (2.0, 13.0)	9.4 ± 8.0 6.5 (3.0, 17.5)
Entheses chronicity score (0–210)	26.9 ± 13.2 26.0 (17.0, 36.0)	29.7 ± 13.7 28.0 (21.0, 39.5)	23.1 ± 11.5 23.0 (15.0, 30.75)

Values are mean ± standard deviation, median (quartile (Q)_1_, Q_3_), or n (%). *Active US enthesitis *Doppler positive hypoechoic and/or thickened enthesis < 2 mm from bone, *CDU* color Doppler ultrasound, *SMI* smooth microvascular imaging, *grade 1 *< 2 punctiform Doppler signals with no confluent Doppler signals*, A* < 2 mm from cortical bone, *B* outside the enthesis, *Doppler score upper/lower extremity *summarized color Doppler score in examined entheses in the upper/lower extremity, *Total Doppler score *summarized Doppler grades in all examined entheses, *Entheses chronicity score *summarized score including enthesophytes size/number, erosions size/number and calcifications

### Reliability test

Intra-observer reliability test regarding Doppler grades and enthesophyte measurements showed ICC 0.995, 95% CI 0.991- 0.997 and ICC 0.998, 95% CI 0.996–0.999, respectively.

### Prevalence of entheseal vascular changes and relation to symptoms

**Fig. 2:**
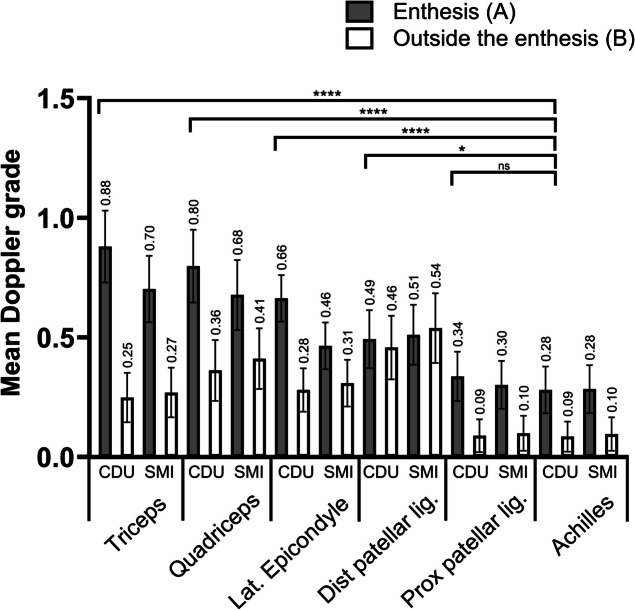
Mean color Doppler grade at six different anatomical sites in a total of 1692 entheses. Legend: Values are mean sum of semiquantitative grading between 0–3 on bilateral examinations at each anatomical location. Whiskers show 95% confidence interval. Wilcoxon signed-rank test with Bonferroni correction was used for pairwise comparisons. *CDU* color Doppler ultrasound, *SMI* smooth microvascular imaging, *Enthesis *(**A**) < 2 mm from cortical bone, *Outside the enthesis *(**B**) tendon proximal to the enthesis. **** *p* < 0.0001, * *p* < 0.05, *ns* non-significant

**Fig. 3:**
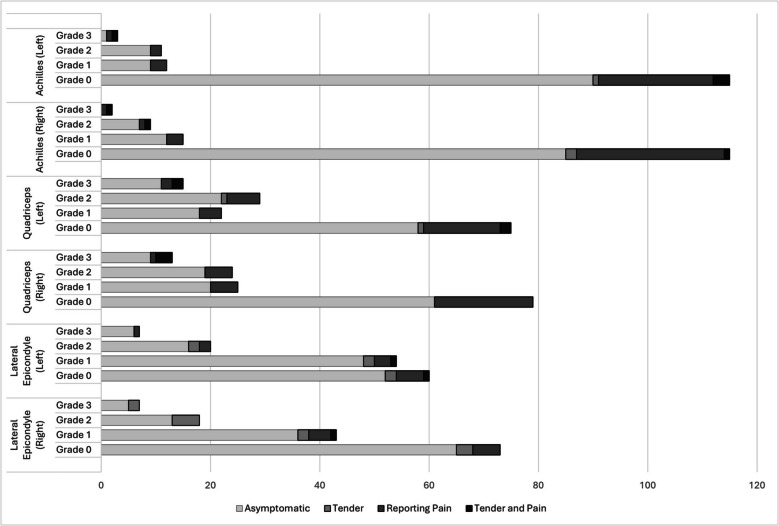
Entheseal Doppler grades and relation to symptoms and signs of enthesitis. Legend: The combined columns in each cluster include a total of 141 patients. Doppler grades are based on maximum values measured in, or outside the enthesis (A or B) using color Doppler ultrasound (CDU). *Tender* positive clinical enthesitis test, *Reporting pain* patient-reported focal pain for more than three months registered on a mannequin. *Asymptomatic* absence of reported focal pain and negative clinical enthesitis test

Figure 2 depicts mean Doppler grade based on bilateral scoring, using CDU and SMI in all examined sites. The mean CDU grades in the entheses (A) were significantly higher in the triceps (0.88), the lateral epicondyle (0.66), and the quadriceps (0.80) compared to the Achilles entheses (0.28) (*p* < 0.0001). Figure 3 presents data on how entheseal CDU grades relate to symptoms and signs of enthesitis. This analysis accounted for findings in, or outside the entheses (A or B). Asymptomatic Doppler grade 2 or 3 were seen in 28 (19.9%) and 22 (15.6%) of the right and left quadriceps entheses, respectively. The corresponding numbers in the right and left Achilles tendons were 7 (5.0%) and 10 (7.1%) (Fig. 3). A full presentation of the CDU grades in all examined entheses and their relation to symptoms is provided in Supplementary Table S1 and Table S2, Additional File 2. Triceps tendons are not included in the clinical enthesitis indices and are therefore absent in Fig. 3 and the Supplementary Tables. As seen in Table 3, 179 of 251 (71.3%) entheses with CDU grade ≥ 2 were asymptomatic. However, the percentage of Doppler grade ≥ 2 scores (A or B) was higher in the tender entheses, 47.0%, compared to the asymptomatic entheses, 16.2%.

**Table 3:** Doppler grades in 1410 individual entheses in relation to symptoms

	**Entheses with Doppler grade ≤ 1** *n* (%)	**Entheses with Doppler grade ≥ 2** *n* (%)	**Total ***n* (%)
**Tender**	35 (53.0)	31 (47.0)	66 (100)
**Non-tender**	1124 (83.6)	220 (16.4)	1344 (100)
**Asymptomatic**	927 (83.8)	179 (16.2)	1106 (100)

Included in this analysis were color Doppler ultrasound (CDU) data from the lateral humeral epicondyle, the quadriceps, proximal and distal patellar ligament, and the Achilles tendon. Maximum Doppler grade in, or outside the enthesis was used at each anatomical location. *Asymptomatic* absence of reporting focal pain on a mannequin, and no tenderness on physical examination

### Factors influencing entheseal vascular changes

#### Bivariate analyses

Bivariate correlations were explored between entheseal CDU scores in different groups of entheses, clinical indices, and laboratory markers, as shown in the correlation matrix, Supplementary Table S3, Additional File 2. No correlation was found between CDU scores (A or A + B) and ASDAS or inflammatory biomarkers, such as neutrophil granulocytes, CRP, and ESR. Doppler signals in different pairs of entheses (A) correlated weakly to moderately with one another (*r* = 0.18–0.24, *p* < 0.01), and a weak correlation was seen between SPARCC enthesitis scores and total CDU scores (A) (*r* = 0.28, *p* < 0.01) (Supplementary Table S3, Additional File 2).

**Fig. 4:**
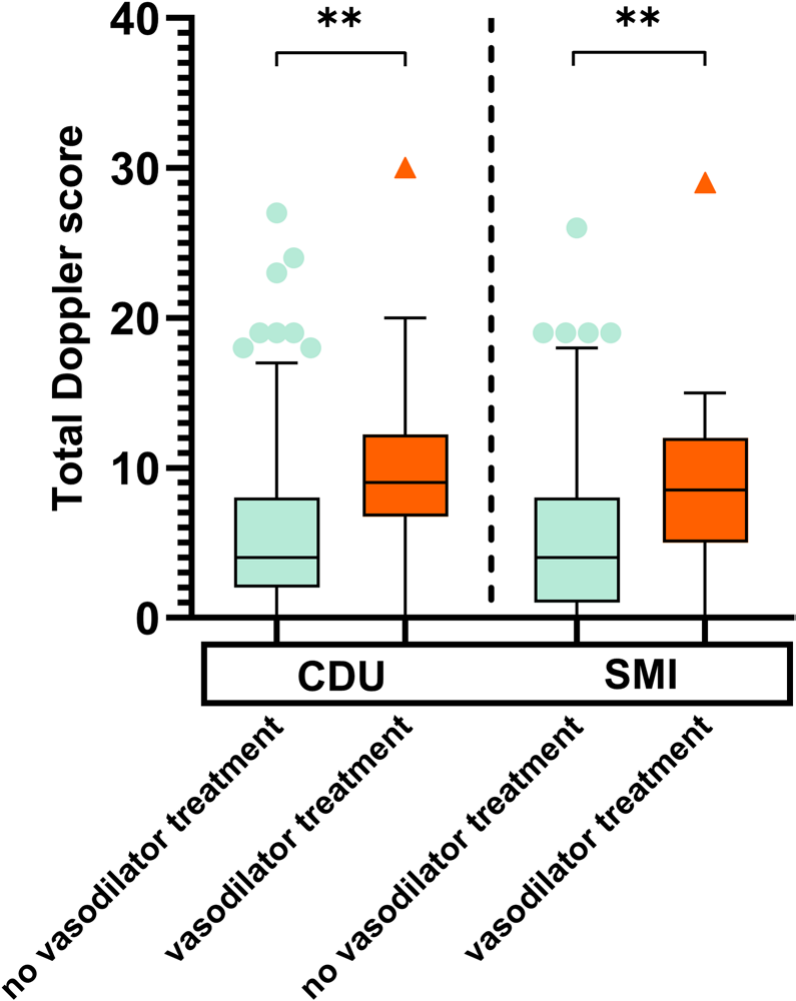
The effect of vasodilator therapy on entheseal Doppler scores. Legend: Total Doppler score is based on summarized Doppler grades measured in the entheses (**A**) in all examined locations using color Doppler ultrasound (CDU) and smooth microvascular imaging (SMI). Mann–Whitney U-test: *p* < 0.005 (CDU), *p* < 0.003 (SMI). Whiskers mark the lowest/highest value within the 1.5 interquartile range beneath/above quartiles 1 and 3. ** *p* < 0.01

As seen in Fig. 4, the effect of vasodilator treatment on entheseal total Doppler scores (A) was tested and revealed higher Doppler scores in individuals treated with vasodilator treatments (*p* < 0.005 (CDU) and *p* < 0.003 (SMI)).

#### Multivariable linear regression analyses

In a further investigation, multivariable linear regression models were explored using log-transformed total Doppler scores (A) as the outcome (Table 4). In model 1, age (B (95% CI) = 0.01 (0.00; 0.01), *p* = 0.004), daily intake of NSAIDs (0.15 (0.00; 0.30), *p* = 0.048) and use of vasodilator drugs (0.16 (0.01; 0.32), *p* = 0.041) were associated with increased total Doppler scores. However, neither ASDAS nor bDMARD treatment were associated with the outcome. These results were uniform irrespective of the Doppler application used.

Sensitivity analyses of model 1 were performed with consistent results. This includes switching the outcome to log-transformed total Doppler score measured in, and outside the enthesis (A + B), and adjusting for the use of ACE- inhibitors and ARBs, with or without diuretics. (Model 3–5, Supplementary Table S4, Additional File 2). In model 3, Supplementary Table S4, the effects of age (0.01 (0.00;0.01), *p* = 0.010), daily NSAIDs (0.18 (0.02;0.35), *p* = 0.029) and vasodilator drugs (0.26 (0.09; 0.43), *p* = 0.003) were similar.

In model 2, Table 4, the influence of longitudinal factors on entheseal Doppler scores was explored. Here, only the entheses chronicity score was associated with the outcome (0.01 (0.01; 0.02), *p* < 0.001).

**Table 4:** Multivariable linear regression analyses exploring influencing factors of a composite total Doppler score

**Current influences**				
	**Model 1** **CDU**		**Model 1** **SMI**	
**R**^**2**^	0.19		0.19	
	**B** (95% CI)	***p*-value**	**B** (95% CI)	***p*-value**
Constant	0.70 (0.20; 1.19)	0.006	0.43 (−0.13; 0.99)	0.13
Age	0.01 (0.00; 0.01)	**0.004**	0.01 (0.00; 0.01)	**0.004**
BMI	−0.01 (−0.03; 0.01)	0.23	−0.01 (−0.03; 0.01)	0.22
Male Sex	−0.06 (−0.17; 0.06)	0.33	−0.01 (−0.14; 0.12)	0.87
ASDAS	−0.03 (−0.11; 0.04)	0.38	0.02 (−0.07; 0.10)	0.68
Physical activity	−0.03 (−0.15; 0.09)	0.66	0.00 (−0.14; 0.14)	1.0
NSAIDs, daily	0.15 (0.00; 0.30)	**0.048**	0.17 (0.00; 0.34)	**0.048**
bDMARD treatment	−0.10 (−0.22; 0.02)	0.09	−0.09 (−0.22; 0.05)	0.20
Vasodilator drug treatment	0.16 (0.01; 0.32)	**0.041**	0.20 (0.02; 0.37)	**0.028**
**Previous influences**				
	**Model 2 CDU**		**Model 2 SMI**	
**R**^**2**^	0.27		0.24	
	**B** (95% CI)	***p*-value**	**B** (95% CI)	***p*-value**
Constant	0.67 (0.19; 1.15)	0.007	0.45 (−0.11; 1.00)	0.11
Age	0.00 (−0.01; 0.01)	0.74	0.00 (−0.01; 0.01)	0.53
Male Sex	−0.13 (−0.26; 0.02)	0.05	−0.09 (−0.25; 0.06)	0.22
Mean BMI 2009- 2022	−0.01 (−0.03; 0.00)	0.10	−0.01 (−0.03; 0.00)	0.13
Mean ASDAS 2009–2022	0.04 (−0.05; 0.12)	0.40	0.08 (−0.02; 0.18)	0.10
Mean ESR 2009–2022	0.00 (−0.01; 0.01)	0.60	−0.00 (−0.01; 0.01)	0.84
Mean CRP 2009–2022	−0.01 (−0.02; 0.00)	0.06	−0.01 (−0.02; 0.01)	0.26
Median occupational physical workload	0.03 (−0.02; 0.09)	0.24	0.01 (−0.05; 0.08)	0.67
Entheses chronicity score	0.01 (0.01;0.02)	**< 0.001**	0.01 (0.01; 0.02)	**< 0.001**

Model 1 explores current influences on total Doppler scores in the entheses (A), while Model 2 investigate the contribution of previous events. The dependent variables are log-transformed and based on CDU or SMI measurements. Highlighted in bold are *p* < 0.05. *CDU* color Doppler ultrasound, *SMI* smooth microvascular imaging, *R*^*2*^ coefficient of determination*, B* unstandardized regression coefficient*, CI* confidence interval, *BMI* body mass index, *ASDAS* Ankylosing Spondylitis Disease Activity Score, *NSAID* non-steroidal anti-inflammatory drug, *bDMARD* biological disease-modifying anti-rheumatic drug, * Entheses chronicity score* summarized score based on presence of enthesophytes, erosions and calcifications

## Discussion

In this study of 141 patients with long-standing r-axSpA, no association was found between Doppler signals in peripheral entheses and ASDAS. Entheseal neovascular changes were common and mostly asymptomatic. The highest Doppler scores were seen in the triceps, whereas scores were low in the Achilles enthesis. A weak bivariate correlation was detected between the entheseal Doppler scores and the SPARCC enthesitis index. Moreover, multivariable linear regression models revealed that higher age and use of vasodilator drugs were related to increased entheseal total Doppler scores.

The percentage of patients with at least one entheseal Doppler signal was substantially higher in the present study compared to previous reports [11, 28, 29]. Our results may be explained by a high sensitivity of Doppler applications, an increased number of examined entheses, and a high mean age of study participants compared to prior studies. CDU was more sensitive than SMI to detect single Doppler signals in deeper structures close to bone, despite identical Doppler frequency settings. Since these observations were not consistent in more superficial tissue, and because SMI has previously been shown to be more sensitive than CDU [30, 31], we suspect that the configuration of the SMI frequency may play a role. However, since comparative studies on Doppler applications in the enthesitis field are lacking, the optimal use of the SMI application remains unclear [32].

The reported occurrence of Doppler signals in individual entheses varies in previous studies. In a study on r-axSpA patients, a low prevalence of Doppler signals was found in the Achilles entheses [29] in accordance with our findings. In a publication by Di Matteo and colleagues on lower limb entheses in axSpA patients, Doppler findings in the Achilles tendon were, however, relatively prevalent [11]. Moreover, we identify a high frequency of inflammatory changes in the elbow entheses. Previous documentation of Doppler findings in the triceps of r-axSpA patients is limited. However, one study on SpA patients reported a high prevalence of Doppler signals at the triceps and lateral epicondyle entheses, compared to both lower extremity entheses and healthy controls [33]. The pronounced degree of Doppler signals in the triceps aligns with a separate investigation on patients with psoriasis [34]. Furthermore, the frequent detection of Doppler signals at the lateral epicondyle in the current study is in agreement with previous research on axSpA, which also demonstrated significant differences relative to non-SpA patients [35, 36].

Differences were found amidst various anatomical locations regarding the agreement between clinical enthesitis and Doppler findings. The lowest frequency of asymptomatic Doppler signals was found in the proximal patellar ligament and in the Achilles tendon. These results partly align with a recent publication showing a high agreement between PD and physical evaluation in the proximal patellar ligament but, a low agreement was seen in the Achilles tendon [14]. We interpret the inconsistencies among studies on entheseal Doppler findings in relation to clinical examination arise from variations in age, disease phenotypes in cohorts, and US machine sensitivity. The correlation between clinical enthesitis indices and Doppler scores was weak, as previously reported [37, 38]. The only index that correlated weakly with Doppler signals was the SPARCC enthesitis index, which is plausible given the composition of entheses examined with US. We interpret that the discrepancy between clinical presentation and US findings stems from the mutual non-specificity of clinical enthesitis tests and entheseal ultrasound findings [6, 9, 39].

Previous studies on explanatory factors of entheseal Doppler signals in r-axSpA are scarce. In our multivariable regression model, patient age influenced Doppler scores, which is coherent with a previous study on healthy individuals by Bakirci et al. [9]. Disease activity did not influence entheseal Doppler scores in our analyses, which to some extent contradict findings of a previous report [11]. However, our results are fully in line with two previous studies by Bandinelli et al., that did not identify any correlations between entheseal PD scores and various clinical disease activity outcome measures in cohorts with IBD and early PsA [12, 13].

BDMARD therapy was not associated with Doppler scores, which is somewhat surprising given its known effect on active enthesitis [40]. However, the low mean disease activity and higher age of patients in our cohort, who were not recently initiated on bDMARD treatment, may limit the ability to detect an association between Doppler scores and bDMARD therapy. Interestingly, BMI was not associated with the total Doppler score, consistent with a study on patients with metabolic syndrome [41]. This finding was unexpected since obesity has been shown to play a role in both the activity and progression of the disease [26, 42]. Furthermore, we performed a secondary analysis to investigate the hypothesis that entheseal neovascular changes are affected by inflammatory and biomechanical stressors over extended periods. While neither longitudinal measurements of inflammation nor disease activity were linked to the outcome, a summarized chronicity score showed an association with the total Doppler score. Indeed, the connection between inflammatory and structural entheseal lesions is to be expected [9]. However, because most Doppler signals were asymptomatic, and there was no association with disease activity or systemic inflammation, our findings suggest that neovascular entheseal changes may be present for long periods.

To the best of our knowledge, the effect of vasodilator drugs such as CCBs on entheseal Doppler signals is a novel finding and, hence, not mentioned in the European Alliance of Associations for Rheumatology (EULAR) recommendations for reporting US studies in rheumatic and musculoskeletal disorders [43]. Since this effect was not seen with ACE inhibitor/ARB treatment, we hypothesize that an increase in capillary hydrostatic pressure caused by CCBs may influence the results [44]. Finally, reported daily NSAIDs were also positively associated with entheseal Doppler scores. We interpret this somewhat unexpected finding to be caused by underlying enthesitis driving NSAID use rather than an actual drug effect. This corresponds to the current finding of a weak but significant correlation between Doppler scores and SPARCC enthesitis index. The effect of NSAID treatment on Doppler abnormalities has been inconsistently reported, and the intervention study by Xu et al. on hand osteoarthritis partly aligns with our findings [45, 46]. In analyses of clinical signs and symptoms and the relationship with Doppler signals, changes outside of the enthesis were considered. This is because imaging findings slightly proximal to the consensus-based 2 mm margin may also be important in this context [47].

Limitations of the study include the inborn operator-dependent nature of ultrasound imaging and the cross-sectional type of the ultrasound data. To increase the validity and reliability of the sonographer, educational efforts were made before study start. Furthermore, the reliability test showed excellent agreement. Despite these efforts and long experience of the study sonographer, it is still possible that subjective factors affect the data. In addition, the clinical assessor was not blinded to the imaging findings, which may have introduced a potential source of bias. Since SMI was consistently measured after CDU by the same investigator, comparisons between these applications should be interpreted with caution. Moreover, a limitation of the study is the relatively low number of tender entheses, which restricts analyses of the agreement between clinical enthesitis and Doppler findings at different anatomical sites. Among the tender entheses, there was a trend toward higher CDU scores on the right side, suggesting that biomechanical loading may influence our data. However, we did not exclude patients with suspected sports-related tendinopathy, such as lateral epicondylitis, as it is difficult to distinguish disease-related changes from those caused by overuse conditions [48]. Strengths of the study include the use of one US machine only with fixed machine settings and stable ambient conditions throughout the study. Furthermore, all Doppler examinations were performed using dual Doppler applications which enable sensitivity analyses. Finally, a strength of the study is the access to highly detailed, longitudinal data on a cohort of long-standing r-axSpA. The size of the cohort and the diversity regarding age and disease severity promote the generalizability of the study results. To our knowledge, previous studies on entheses of older individuals with r-axSpA are non-existent.

We did not find an association between entheseal Doppler signals and clinically available inflammatory biomarkers. Because these markers may not be sensitive enough, future studies could explore associations between entheseal changes and other proinflammatory molecules. The finding that vasodilator drugs increase entheseal Doppler scores is potentially clinically important and warrants further research. Indeed, since this confounder also may affect US examination of joints.

## Conclusions

Doppler signals in peripheral entheses of patients with long-standing r-axSpA were common, mostly asymptomatic, and not associated with disease activity. The varying prevalence of asymptomatic Doppler signals in different anatomical sites and the effect of age and vasodilator drugs need to be considered when assessing entheses using ultrasound. Ultimately, these results emphasize the importance of integrating clinical presentation when interpreting imaging findings in the entheses.

## Supplementary information

Additional file 1: Figure S1. Representative image examples of Doppler grading. Figure S2, Definition of the enthesis.

Additional file 2: Table S1. Frequency of Doppler findings in tender enthuses. Table S2, Frequency of Doppler findings in non-tender enthuses. Table S3, Correlation matrix. Table S4, Sensitivity analyses of multivariable linear regression.

## Data Availability

Due to Swedish legislation (the Personal Data Act), the data set analyzed in this study is not publicly available, but a limited data set that supports the main findings can be made available upon reasonable request.

## Abbreviations

(A): Enthesis < 2 mm from the bony cortex
ACE: Angiotensin-converting enzyme
ARB: Angiotensin receptor blocker
AS: Ankylosing spondylitis
ASAS: Assessment of SpondyloArthritis international Society
ASDAS: Ankylosing Spondylitis Disease Activity Score
(B): Outside the enthesis
B: Unstandardized regression coefficient
BASDAI: Bath Ankylosing Spondylitis Disease Activity Index
BASFI: Bath Ankylosing Spondylitis Functional Index
BASMI: Bath Ankylosing Spondylitis Metrology Index
bDMARDs: Biological disease-modifying antirheumatic drugs
BMI: Body mass index
CCB: Calcium channel blocker
CDU: Color Doppler ultrasound
EULAR: European alliance of associations for rheumatology
ESR: Erythrocyte sedimentation rate
HLA-B27: Human leukocyte antigen B27
Hs-CRP: High-sensitivity c-reactive protein
ICC: Intraclass correlation
LEI: Leeds enthesitis index
LOAS: Long-term outcome ankylosing spondylitis
MASES: Maastricht ankylosing spondylitis enthesitis score
mSASSS: Modified Stoke ankylosing spondylitis spinal score
ns: Non-significant
NSAID: Non-steroidal anti-inflammatory drug
OMERACT: Outcomes measures in rheumatology
Ρ: Pearson correlation coefficient
PD: Power Doppler
Q: Quartile
R^2^: Coefficient of determination
r-axSpA: Radiographic axial spondyloarthritis
SMI: Smooth microvascular imaging
SpA: Axial spondyloarthritis
SPARCC: Spondyloarthritis research consortium of Canada
SD: Standard deviation
US: Ultrasound
