# Supplementary material for: Entheseal Doppler signals in ultrasound are associated with vasodilator drugs and age in patients with radiographic axial spondyloarthritis
Source: Arthritis Res Ther. 2025 Jul 14;27:149. doi: 10.1186/s13075-025-03614-8 (PMC12261538; doi:10.1186/s13075-025-03614-8)
Supplement: Supplementary file 1 — Additional file 1: Figure S1. Representative image examples of Doppler grading. Figure S2, Definition of the enthesis. [file 13075_2025_3614_MOESM1_ESM.docx]

**Additional file 1: Supplementary Figures**

**Supplementary Figure S1**

**Title:** Representative image examples of entheseal Doppler grade 0 to 3

**
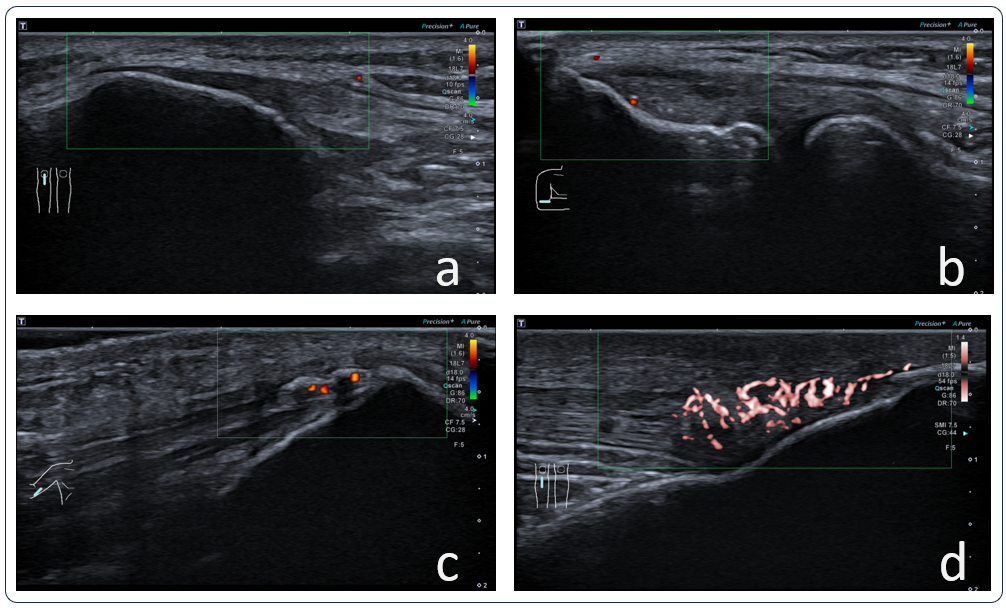
**

Doppler signals were scored semi-quantitatively from 0 to 3. In picture a, the enthesis of the proximal patellar ligament is seen without Doppler signals, graded as a 0. In b, the common extensor enthesis at the lateral humeral epicondyle is shown with < 2 punctiform Doppler signals with no confluent Doppler signal and thus graded as 1. In c, the distal triceps enthesis is graded as 2, which means 2-4 punctiform Doppler signals or 1 confluent Doppler signal. In picture d, the distal insertion of the patellar ligament is displayed with > 4 punctiform Doppler signals or > 1 confluent Doppler signals and therefore graded as 3. In images a-c, color Doppler ultrasound (CDU) is shown, while d illustrates the smooth microvascular imaging (SMI) application.

**Supplementary Figure S2**

**Title**: Definition of the enthesis as seen on ultrasound

**
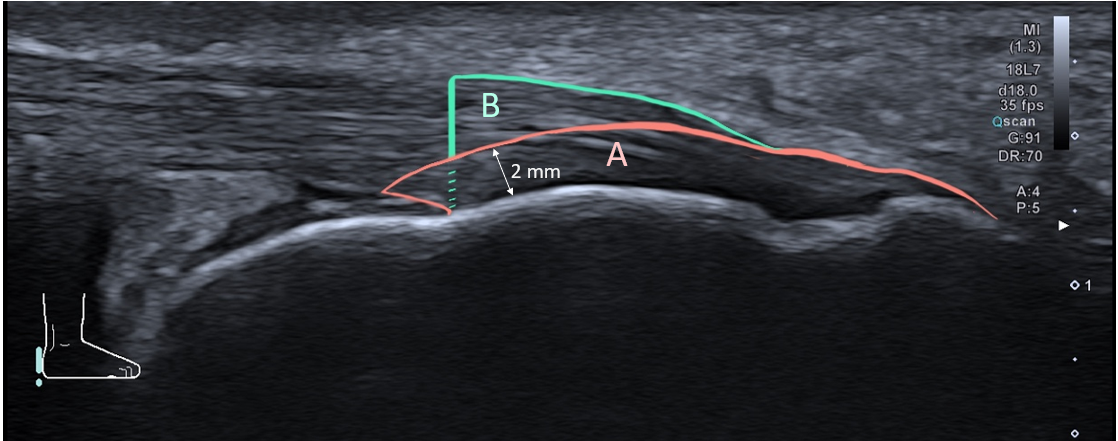
**

Doppler scores were graded within a 2 mm margin from cortical bone, defined as the enthesis (A), and proximal to this margin (B), defined as outside the enthesis.
