# Supplementary material for: Entheseal Doppler signals in ultrasound are associated with vasodilator drugs and age in patients with radiographic axial spondyloarthritis
Source: Arthritis Res Ther. 2025 Jul 14;27:149. doi: 10.1186/s13075-025-03614-8 (PMC12261538; doi:10.1186/s13075-025-03614-8)
Supplement: Supplementary file 2 — Additional file 2: Table S1. Frequency of Doppler findings in tender enthuses. Table S2, Frequency of Doppler findings in non-tender enthuses. Table S3, Correlation matrix. Table S4, Sensitivity analyses of multivariable linear regression. [file 13075_2025_3614_MOESM2_ESM.docx]

**Additional file 2: Supplementary Tables**

**Supplementary Table S1**

| **Frequency of Doppler findings in 66 tender entheses** | | | | | | |
| --- | --- | --- | --- | --- | --- | --- |
|  | **Color Doppler grade**  Mean ± SD  Median  (Q1; Q3) | **Tender**  **grade 0**  **Doppler findings**  n (% of all grade 0 findings) | **Tender**  **grade 1**  **Doppler findings**  n (% of all grade 1 findings) | **Tender**  **grade 2**  **Doppler findings**  n (% of all grade 2 findings) | **Tender**  **grade 3**  **Doppler findings**  n (% of all grade 3 findings) | **Tender**  **grade ≥ 2**  **Doppler findings**  n (% of all tender entheses) |
| **Right lateral epicondyle** | 1.46 ± 1.05  2.0 (0.5; 2.0) | 3 (4.1) | 3 (7.0) | 5 (27.8) | 2 (28.6) | 7 (53.8) |
| **Left lateral epicondyle** | 0.88 ± 0.84  1.0 (0.0; 1.75) | 3 (5.0) | 3 (5.6) | 2 (10.0) | 0 (0.0) | 2 (25.0) |
| **Right quadriceps** | 3.0 ± 0.0  3.0 (3.0; 3.0) | 0 (0.0) | 0 (0.0) | 0 (0.0) | 3 (23.1) | 3 (100) |
| **Left quadriceps** | 1.33 ± 1.51  1.0 (0; 3.0) | 3 (4.0) | 0 (0.0) | 1 (3.4) | 2 (13.3) | 3 (50.0) |
| **Right prox. patellar ligament** | 1.33 ± 1.51  1.0 (0.0; 3.0) | 3 (2.7) | 0 (0.0) | 1 (11.1) | 2(33.3) | 3 (50.0) |
| **Left prox. patellar ligament** | 0.86 ± 1.22  0.0 (0.0; 2.0) | 4 (3.5) | 1 (7.7) | 1 (10.0) | 1 (20.0) | 2 (28.6) |
| **Right distal patellar ligament** | 1.67 ± 1.37  2.0 (0; 3.0) | 2 (2.1) | 0 (0.0) | 2 (10.0) | 2 (22.2) | 4 (66.7) |
| **Left distal patellar ligament** | 1.29 ± 1.25  2.0 (0.0; 2.0) | 3 (3.4) | 0 (0.0) | 3 (11.1) | 1 (14.3) | 4 (57.1) |
| **Right Achilles** | 1.0 ± 1.41  0.0 (0; 2.5) | 3 (2.6) | 0 (0.0) | 1 (11.1) | 1 (50.0) | 2 (40.0) |
| **Left Achilles** | 0.60 ± 1.34  0.0 (0.0; 1.5) | 4 (3.5) | 0 (0.0) | 0 (0.0) | 1 (33.3) | 1 (20.0) |

Color Doppler ultrasound (CDU) was used and the maximum Doppler grade either in the enthesis (A), or outside the enthesis (B) was registered. *SD* Standard deviation, *Q* Quartile, *Tender* tender on palpation, *Asymptomatic* absence of reported focal pain, and negative clinical enthesitis test, *Grade 0* no Doppler signal, *Grade 1* < 2 punctiform Doppler signals with no confluent Doppler signal, *Grade 2* 2-4 punctiform Doppler signal or 1 confluent Doppler signal, *Grade 3* > 4 punctiform Doppler signals or > 1 confluent Doppler signal

**Supplementary Table S2**

| **Frequency of Doppler findings in 1344 non-tender entheses** | | | | | | | |
| --- | --- | --- | --- | --- | --- | --- | --- |
|  | **Color Doppler grade**  Mean ± SD  Median  (Q1; Q3) | **Non-**  **tender**  **grade 0**  **Doppler findings**  n (% of all  grade 0 findings) | **Non-tender**  **grade 1**  **Doppler findings**  n (% of all  grade 1 findings) | **Non-tender**  **grade 2**  **Doppler findings**  n (% of all  grade 2 findings) | **Non-tender**  **grade 3**  **Doppler findings**  n (% of all  grade 3 findings) | **Non-**  **Tender**  **grade ≥ 2 Doppler findings**  n (% of  all non-tender entheses) | **Asympt-**  **omatic entheses**  **with grade 0**  n (% of  all asympt-  omatic entheses) |
| **Right lateral epicondyle** | 0.63 ± 0.82  0.0 (0.0; 1.0) | 70 (95.9) | 40 (93.0) | 13 (72.2) | 5 (71.4) | 18 (14.1) | 65 (54.6) |
| **Left lateral epicondyle** | 0.81 ± 0.86  1.0 (0.0; 1.0) | 57 (95.0) | 51 (94.4) | 18 (90.0) | 7 (100) | 25 (18.8) | 52 (42.6) |
| **Right quadriceps** | 0.75 ± 0.99  0.0 (0.0; 1.25) | 79 (100) | 25 (100) | 24 (100) | 10 (76.9) | 34 (24.6) | 61 (56.0) |
| **Left**  **quadriceps** | 0.87 ± 1.06  0.0 (0.0; 2.0) | 72 (96.0) | 22 (100) | 28 (96.6) | 13 (86.7) | 41 (30.4) | 58 (53.2) |
| **Right prox. patellar ligament** | 0.33 ± 0.72  0.0 (0.0; 0.0) | 107 (97.3) | 16 (100) | 8 (88.9) | 4 (66.7) | 12 (8.9) | 87 (80.6) |
| **Left prox. patellar ligament** | 0.31 ± 0.73  0.0 (0.0; 0.0) | 109 (96.5) | 12 (92.3) | 9 (90.0) | 4 (80.0) | 13 (9.7) | 90 (82.6) |
| **Right distal patellar ligament** | 0.55 ± 0.91  0.0 (0.0; 1.0) | 93 (97.9) | 17 (100) | 18 (90.0) | 7 (77.8) | 25 (18.5) | 74 (68.5) |
| **Left distal patellar ligament** | 0.63 ± 0.93  0.0 (0.0; 1.0) | 86 (96.6) | 18 (100) | 24 (88.9) | 6 (85.7) | 30 (22.4) | 71 (65.1) |
| **Right Achilles** | 0.25 ± 0.59  0.0 (0.0; 0.0) | 112 (97.4) | 15 (100) | 8 (88.9) | 1 (50.0) | 9 (6.6) | 85 (81.7) |
| **Left Achilles** | 0.29 ± 0.68  0.0 (0.0; 0.0) | 111 (96.5) | 12 (100) | 11 (100) | 2 (66.7) | 13 (9.6) | 90 (82.6) |

Color Doppler ultrasound (CDU) was used and the maximum Doppler grade either in the enthesis (A), or outside the enthesis (B) was registered. *SD* Standard deviation, *Q* Quartile, *Tender* tender on palpation, *Asymptomatic* absence of reported focal pain, and negative clinical enthesitis test, *Grade 0* no Doppler signal, *Grade 1* < 2 punctiform Doppler signals with no confluent Doppler signal, *Grade 2* 2-4 punctiform Doppler signal or 1 confluent Doppler signal, *Grade 3* > 4 punctiform Doppler signals or > 1 confluent Doppler signal

**Supplementary Table S3**

| **Correlation matrix of clinical indices, inflammatory biomarkers, and entheseal Doppler scores** | | | | | | | | | | | | | | | | | | | |
| --- | --- | --- | --- | --- | --- | --- | --- | --- | --- | --- | --- | --- | --- | --- | --- | --- | --- | --- | --- |
|  | **Age** | **BMI** | **Total CDU**  **in A** | **Total CDU in A + B** | **Lateral epicondyle**  **CDU in A** | **Triceps**  **CDU in A** | **Quadri-**  **ceps**  **CDU in A** | **Proximal patellar ligament**  **CDU in A** | **Distal patellar ligament CDU in A** | **Achilles CDU in A** | **ASDAS** | **BASDAI** | **BASFI** | **LEI** | **SPARC** | **MASES** | **Neutro-**  **phils** | **ESR** | **Hs CRP** |
| **Age** | 1 |  |  |  |  |  |  |  |  |  |  |  |  |  |  |  |  |  |  |
| **BMI** | 0.12 | 1 |  |  |  |  |  |  |  |  |  |  |  |  |  |  |  |  |  |
| **Total CDU in A** | 0.29** | -0.04 | 1 |  |  |  |  |  |  |  |  |  |  |  |  |  |  |  |  |
| **Total CDU in**  **A + B** | 0.21* | -0.05 | 0.94** | 1 |  |  |  |  |  |  |  |  |  |  |  |  |  |  |  |
| **Lateral epicondyle**  **CDU in A** | 0.01 | -0.22* | 0.45** | 0.42** | 1 |  |  |  |  |  |  |  |  |  |  |  |  |  |  |
| **Triceps CDU in A** | 0.41** | 0.09 | 0.75** | 0.69** | 0.19* | 1 |  |  |  |  |  |  |  |  |  |  |  |  |  |
| **Quadriceps CDU in A** | 0.27** | -0.06 | 0.78** | 0.71** | 0.24** | 0.46** | 1 |  |  |  |  |  |  |  |  |  |  |  |  |
| **Proximal patellar ligament CDU in A** | 0.09 | -0.01 | 0.67** | 0.64** | 0.13 | 0.38** | 0.46** | 1 |  |  |  |  |  |  |  |  |  |  |  |
| **Distal patellar ligament CDU in A** | 0.20* | -0.04 | 0.75** | 0.71** | 0.18* | 0.47** | 0.42** | 0.60** | 1 |  |  |  |  |  |  |  |  |  |  |
| **Achilles CDU in A** | 0.07 | 0.03 | 0.62** | 0.63** | 0.24** | 0.34** | 0.41** | 0.09 | 0.31** | 1 |  |  |  |  |  |  |  |  |  |
| **ASDAS** | 0.19* | 0.19* | 0.13 | 0.15 | -0.04 | 0.17* | -0.02 | 0.22* | 0.21** | -0.00 | 1 |  |  |  |  |  |  |  |  |
| **BASDAI** | 0.14 | 0.15 | 0.10 | 0.11 | -0.02 | 0.14 | -0.10 | 0.15 | 0.19* | 0.06 | 0.88** | 1 |  |  |  |  |  |  |  |
| **BASFI** | 0.43** | 0.39** | 0.13 | 0.12 | 0.01 | 0.24** | 0.02 | 0.11 | 0.15 | -0.03 | 0.64** | 0.61** | 1 |  |  |  |  |  |  |
| **LEI** | -0.01 | 0.14 | 0.13 | 0.15 | 0.08 | 0.14* | 0.08 | 0.05 | 0.10 | 0.09 | 0.35** | 0.32** | 0.30** | 1 |  |  |  |  |  |
| **SPARCC** | 0.10 | 0.13 | 0.28** | 0.29** | 0.09 | 0.22** | 0.17 | 0.22** | 0.24** | 0.21* | 0.43** | 0.45** | 0.36** | 0.80** | 1 |  |  |  |  |
| **MASES** | 0.05 | 0.24** | 0.15 | 0.101 | 0.06 | 0.21* | 0.00 | 0.17* | 0.11 | 0.07 | 0.37** | 0.40** | 0.30** | 0.52** | 0.62** | 1 |  |  |  |
| **Neutrophils** | 0.16 | 0.07 | 0.04 | -0.03 | -0.10 | 0.08 | 0.00 | 0.03 | 0.13 | -0.01 | 0.29** | 0.203* | 0.14 | 0.17* | 0.14 | 0.15 | 1 |  |  |
| **ESR** | 0.13 | 0.07 | 0.12 | 0.15 | -0.21* | 0.23** | 0.07 | 0.15 | 0.05 | 0.11 | 0.21* | 0.09 | 0.10 | -0.07 | 0.04 | 0.07 | -0.00 | 1 |  |
| **Hs CRP** | 0.12 | 0.08 | 0.06 | 0.12 | -0.12 | 0.05 | 0.01 | 0.16 | 0.16 | -0.04 | 0.38** | 0.09 | 0.05 | 0.06 | 0.11 | 0.06 | 0.15 | 0.36** | 1 |

Values are Pearson correlation coefficients. * p <0.05, ** p <0.01. Doppler grades of individual pairs of entheses are mean values of bilateral measurements < 2mm from cortical bone. *BMI* body mass index, *CDU* color Doppler ultrasound, *A* <2 mm from the cortical bone, *B* outside the enthesis, *ASDAS* Ankylosing Spondylitis Disease Activity Score, *BASDAI* Bath Ankylosing Spondylitis Disease Activity Index, *BASFI* Bath Ankylosing Spondylitis Functional Index, *LEI* Leeds Enthesitis Index, *SPARCC* Spondyloarthritis Research Consortium of Canada enthesitis index, *MASES* Maastricht AS Enthesitis Score, *ESR* erythrocyte sedimentation rate, *hs CRP* high sensitivity C-reactive protein.

**Supplementary Table S4**

| **Sensitivity analyses of multivariable linear regression analyses exploring factors influencing entheseal total Doppler scores.** | | | | | | |
| --- | --- | --- | --- | --- | --- | --- |
|  | **Model 3**  **CDU, A + B** | | **Model 4**  **SMI, A + B** |  | **Model 5**  **CDU, A** |  |
| **R^2^** | 0.19 | | 0.20 |  | 0.19 |  |
|  | **B** (95% CI) | **p-value** | **B** (95% CI) | **p-value** | **B** (95% CI) | **p-value** |
| **Constant** | 0.85 (0.31; 1.39) | 0.002 | 0.58 (-0.05; 1.20) | 0.07 | 0.73 (0.21; 1.24) | 0.006 |
| **Age** | 0.01 (0.00; 0.01) | **0.010** | 0.01 (0.00; 0.01) | **0.009** | 0.01 (0.00; 0.01) | **0.010** |
| **BMI** | -0.01 (-0.03; 0.01) | 0.16 | -0.01 (-0.03; 0.01) | 0.17 | -0.01 (-0.03; 0.01) | 0.21 |
| **Male Sex** | -0.05 (-0.17; 0.08) | 0.45 | 0.01 (-0.13; 0.15) | 0.89 | -0.06 (-0.17; 0.06) | 0.32 |
| **ASDAS** | -0.04 (-0.12; 0.05) | 0.41 | 0.02 (-0.08; 0.11) | 0.73 | -0.03 (-0.11; 0.04) | 0.39 |
| **Physical activity** | -0.01 (-0.14; 0.12) | 0.88 | 0.03 (-0.13; 0.18) | 0.74 | -0.02 (-0.15; 0.10) | 0.70 |
| **NSAIDs, daily** | 0.18 (0.02; 0.35) | **0.029** | 0.20 (0.01; 0.39) | **0.041** | 0.15 (-0.01; 0.30) | 0.06 |
| **bDMARD treatment** | -0.06 (-0.19; 0.07) | 0.38 | -0.06 (-0.21; 0.09) | 0.42 | -0.10 (-0.22; 0.02) | 0.09 |
| **Vasodilator drug treatment** | 0.26 (0.09; 0.43) | **0.003** | 0.30 (0.10; 0.50) | **0.003** | 0.16 (0.00; 0.32) | **0.048** |
| **Antihypertensive medication** | NA |  | NA |  | 0.03 (-0.11; 0.17) | 0.67 |

The outcomes of models 3 and 4 are total Doppler score measured in, and outside the entheses (A+B), using color Doppler ultrasound (CDU) and smooth microvascular imaging (SMI), respectively. In model 5, Doppler is measured exclusively in the enthesis (A) using CDU. All outcomes are log-transformed. Highlighted in bold are p < 0.05. *R^2^* coefficient of determination*, B* unstandardized regression coefficient*, CI* confidence interval, *BMI* body mass index, *ASDAS* Ankylosing Spondylitis Disease Activity Score, *physical activity* score based on patient-reported hours of vigorous-intensity physical activity per week: 0 = 0, <0.5 = 1, 0.5-1 = 2, 1-1.5 = 3, 1.5-2 = 4, >2 = 5, *NSAID* non-steroidal anti-inflammatory drug, *bDMARD* biological disease-modifying anti-rheumatic drug, *Vasodilator drug treatment* continuous use of calcium channel blockers (CCBs), glyceryl trinitrate or buprenorphine,  *Antihypertensive medication* continuous use of angiotensin-converting enzyme inhibitors, angiotensin receptor blockers, with or without combined diuretics.
